# *In Vitro* and *In Silico* Antimicrobial Activity against Methicillin-Resistant *Staphylococcus aureus* of Essential Oils from Four Medicinal Plants in Xuan Thuy Mangrove Forest

**DOI:** 10.4014/jmb.2409.09006

**Published:** 2025-02-14

**Authors:** Ngoc Anh Luu Dam, Van Huong Bui, Ton That Huu Dat, Khac Tiep Nguyen, Thanh Tung Nguyen, Quang Quy Duong, Tung Lam Vo, Vladimir V. Titok

**Affiliations:** 1 Vietnam National Museum of Nature, Vietnam Academy of Science and Technology, 18 Hoang Quoc Viet, Cau Giay, Ha Noi, Vietnam; 2 Mientrung Institute for Scientific Research, Vietnam National Museum of Nature, Vietnam Academy of Science and Technology, 321 Huynh Thuc Khang, Hue City, Vietnam; 3 Hanoi University of Pharmacy, 13-15 Le Thanh Tong, Hoan Kiem, Ha Noi, Vietnam; 4 Central Botanical Garden, National Academy of Sciences of Belarus, Minsk, Belarus

**Keywords:** *In vitro*, *in silico*, antimicrobial activity, methicillin-resistant, *Staphylococcus aureus*, essential oils

## Abstract

*Sphagneticola trilobata*, *Vitex rotundifolia*, *Vitex trifolia*, and *Annona glabra* are medicinal plants found in Xuan Thuy mangrove forest that contain essential oils (EOs) and the potential to treat infections. The obtained yields of hydrodistillation essential oil from aerial parts and leaves ranged from 0.01% to 0.12% (v/w) on a fresh-weight basis. Subsequently, *in vitro* assessments indicated that the EOs of *A. glabra*, *S. trilobata*, and *V. trifolia* displayed antibacterial effects against methicillin-resistant *Staphylococcus aureus* (MRSA), with *S. trilobata* exhibiting bactericidal effects at minimum inhibitory concentration (MIC) and minimum bactericidal concentration (MBC) values of 0.2% EO, and *A. glabra* exhibiting bacteriostatic effects with both MIC and MBC values of 0.8% EO. However, the EO of *V. rotundifolia* showed no inhibitory effects against *S. aureus*, even at concentrations of up to 0.8% EO. Furthermore, docking results showed that six compounds exhibited particularly high binding energies (< -7 kcal/mol): *α*-phellandrene, *β*-caryophyllene, *β*-guaiene, alloaromadendrene, bicyclosesquiphellandrene, and bicyclogermacrene. Among these, *α*-phellandrene, *β*-caryophyllene, bicyclosesquiphellandrene, and bicyclogermacrene are the main components in the *S. trilobata* EO; *β*-caryophyllene, *β*-guaiene, and alloaromadendrene are the main components in the *A. glabra* EO; and *β*-caryophyllene and alloaromadendrene are the main components in the *V. trifolia* EO. These findings highlight the antibacterial potential of EOs from mangrove plants; therefore, expanded investigations into EOs and the biological properties of mangrove plants may be a promising strategy for combating antimicrobial resistance.

## Introduction

Antimicrobial resistance (AMR) is a pressing global health issue that threatens the efficacy of antibiotics, leading to increased morbidity and mortality rates. The misuse and overuse of antibiotics in human medicine, veterinary practices, and agriculture have accelerated the emergence of resistant pathogens, creating a "Silent Pandemic" that could surpass other health crises by 2050 [1]. AMR complicates the treatment of infections, resuting in millions of deaths annually and the financial implications of AMR are substantial, with projected losses in the trillions of dollars [1, 2].

*Staphylococcus aureus* has been recognized as one of the most important pathogens for human and animal health. It is implicated in both community-acquired and nosocomial infections. Especially, methicillin-resistant *Staphylococcus aureus* (MRSA), poses a significant threat in healthcare facilities and to public health worldwide due to its resistance to antibiotic treatment, which complicates the management of infections [3]. The prevalence of MRSA infections is alarming, with reports indicating that up to 90% of *S. aureus* infections in certain regions are resistant to standard antibiotics [4]. The presence of MRSA can lead to severe consequences, such as life-threatening sepsis, pneumonia, and increased incidences of myocardial infarction and heart failure among individuals with bloodstream infections [5].

Current methods to target antibiotic-resistant strains focus on disrupting bacterial metabolism, inhibiting efflux pumps, and employing novel therapeutic strategies. However, these approaches face significant limitations that hinder their effectiveness [6-8]. Resistance mechanisms can quickly adapt, rendering metabolic targeting less effective over time. Moreover, the emergence of new efflux systems and mutations can undermine these strategies, complicating treatment efforts. Non-traditional methods such as bacteriophage therapy, anti-virulence strategies, and nanoparticle-based therapies are being explored [6]. However, these approaches often face challenges in transitioning from laboratory settings to clinical applications due to regulatory hurdles and efficacy concerns [9]. To date, the potential of bioactive compounds from medicinal plants, particularly essential oils (EOs), as alternative antimicrobial agents is increasingly recognized in the fight against antibiotic resistance. These natural compounds exhibit unique mechanisms of action that can effectively target multidrug-resistant (MDR) bacteria, offering a promising avenue for new therapeutic strategies [10-13].

Several medical plants in mangrove forests in Vietnam, such as *Sphagneticola trilobata*, *Vitex rotundifolia*, *Vitex trifolia*, and *Annona glabra* contain EOs and the potential to treat infections [14]. *S. trilobata* is cultivated and used by the Vietnamese as a traditional medicine. The methanol extract of *S. trilobata* flower had excellent antioxidant activities [15]. The other two mangrove plants, *V. rotundifolia* and *V. trifolia* are commonly used to treat headaches, fever, diarrhea, hair loss, wound recovery, and other diseases [14, 16]. Pharmacological studies have shown that *Vitex rotundifolia* L. f. and *V. trifolia* L. have a variety of pharmacological activities, such as anti-tumor, analgesic, antipyretic, anti-inflammatory, antioxidant, antibacterial, and estrogen-like activity [17]. In modern clinical settings, these plants are used for treating cold headaches, diarrhea dysentery, irregular menstruation, and other diseases [18]. However, in-depth research on the pharmacological activities of *V. rotundifolia* L. f. and *V. trifolia* L. has not been conducted, and the potential active components still need to be explored. In traditional medicine, another mangrove plant, *A. glabra*, is also used to treat fever, diarrhea, dysentery, asthma, and parasites. This plant also has antispasmodic, laxative, antiemetic, and antidepressant effects [16, 19]. It is a potential source of compounds for cancer therapy, where an alcoholic seed extract has shown anticancer activity [20-22]. The alkaloid, phenolic, cyclopeptide, acetogenin, fatty acid, and essential oil compounds extracted from *A. glabra* have demonstrated biological activity, both *in vivo* and *in vitro* [23], such as against ethanol-induced neurodegeneration in neonatal rats [24], against human cervical cancer cells (HeLa), prostate adenocarcinoma metastatic (PC3), and ovary adenocarcinoma (SKOV3) [25]. Its fruit extract has anticancer effects on lung adenocarcinoma cell line (LU-1) human breast carcinoma (MCF-7) [26]. Additionally, *A. glabra* extracts might be used to treat acute hepatopancreatic necrosis disease (AHNPD) in white-leg shrimp [27].

Due to the high number of variables that can influence the biological activity of individual or mixtures of compounds, it is necessary to conduct a high number of screening tests to verify the biological activities (antibacterial, antioxidant, antitumor, etc.), mechanisms of action, and active concentrations. It would be therefore desirable to develop an *in silico* method that allows obtaining information before conducting the biological tests, to reduce the analysis times and costs. In this paper, the antibacterial effects against MRSA of EOs from four plant species collected in the mangrove forests of Xuan Thuy National Park were investigated. Furthermore, docking research was carried out to determine the interaction ability of compounds in EOs with corresponding target proteins of *S. aureus*. Our results could be useful for further experimental studies that aim to develop and discover new antibacterial agents against *S. aureus*.

## Materials and Methods

### Chemicals and Reagents

Tween 80, Na_2_SO_4_, cation-adjusted Muller Hinton broth 2 (CA-MHB), and tryptic soy agar (TSA) were obtained from Merck (Germany); C7 - C40 Saturated Alkanes Standard was obtained from Sigma-Aldrich (USA). All chemicals and reagents were of analytical grade. The bacterial strain *Staphylococcus aureus* ATCC 33591 was supplied by the Hanoi University of Pharmacy.

### Plant Materials

Aerial parts of *Sphagneticola trilobata* and fresh leaves of three other medicinal plants, *V. rotundifolia*, *V. trifolia*, and *A. glabra*, were collected in mangrove forests at Xuan Thuy National Park in July 2023. The voucher specimens, XT14 – *A. glabra*, XT 48 – *V. rotundifolia*, XT 16 – *V. trifolia*, and XT 30 – *S. trilobata*, were deposited in the Herbarium of Vietnam National Museum of Nature in Ha Noi.

### Essential Oil Extraction

The essential oils were hydrodistilled for 3 h using a Clevenger apparatus as described in Vietnamese Pharmacopoeia V [28]. The obtained essential oil was dried over anhydrous sodium sulfate Na_2_SO_4_ to remove any trace of water and stored in sealed glass vials at 4°C until further analysis.

### Gas Chromatography-Mass Spectrometry (GC-MS)

In the gas chromatography-mass spectrometer (GC-MS) analysis of the essential oil of four medicinal plants (*S. trilobata*, *V. rotundifolia*, *V. trifolia*, and *A. glabra*), a Thermo Scientific Trace 1310 system coupled with a Thermo Scientific ITQ 900 mass spectrometer equipped with a capillary column TG-5MS (30 m × 0.25 mm i.d, 0.25 μm film thickness) was used. The oven temperature was held at 40°C, then programmed to 240°C (held 5 min) at a rate of 4°C/min. Helium was used as the carrier gas at a flow rate of 1.0 ml/min. The injector temperature was 250°C, and the injection volume was 0.1 ml in *n*-hexane, with a split ratio of 1:50. Mass spectra (MS) were obtained in the electron impact mode (70 eV), and the MS data were acquired in scan mode with a mass range of *m/z* 40–450. The identification of the components was made based on the retention index (RI) relative to a homologous series of standard n-alkanes (C7 - C40 Saturated Alkane Standard, Sigma-Aldrich) under identical experimental conditions, MS library search (NIST 14 version 2.2), the Automated Mass Spectral Deconvolution & Identification System (AMDIS_32), and by comparing with MS literature data [29].

### Antimicrobial Activity

The reference strain *S. aureus* ATCC 33591 (gram-positive, methicillin-resistant *S. aureus* - MRSA) was obtained from library stock cultures. Minimum inhibitory concentrations (MICs) of the essential oils were determined by microdilution on the 96-well plate from SPL in cation-adjusted Muller Hinton broth 2 (CA-MHB), from Sigma-Aldrich, following Clinical and Laboratory Standards Institute recommendations [30].

The stock solution of essential oils was emulsified in a solution of 4% Tween 80 in water, and the final concentration of Tween 80 in the antimicrobial assays was 0.002%. Their concentrations were calculated according to the percentage of EOs in the water (%, v/v). The reference standard is meropenem from Sigma-Aldrich, following Clinical and Laboratory Standards Institute recommendations [30]. Tween 80 at a concentration of 0.002% was used as a negative control. Minimum bactericidal concentration (MBC) was determined from MIC tests by subculturing wells with concentrations higher than MIC on tryptic soy agar (TSA), and then counting the colonies after 24 h incubation. In this study, the MBC is the lowest concentration required to kill >99.9% of microorganisms, compared with the initial inoculum (1.5x106 for bacteria). The ratio MBC/MIC is a method used for evaluating the bactericidal activities.

### Molecular Docking Analysis

Four protein targets of *S. aureus* were selected for the docking simulation. These key proteins involved in the replication and virulence of *S. aureus* include tyrosyl-tRNA synthetase (PDB ID: 1JIJ) [31], dihydrofolate reductase (PDB ID: 2W9S) [32], dehydrosqualene synthase (PDB ID: 2ZCQ) [33], and cell division protein FtsZ (PDB ID: 4DXD) [34]. The 3D structures of these proteins were obtained from the Protein Data Bank (https://www.rcsb.org/). MOE2015.10 software was used to refine the protein structures, including steps to add missing hydrogen atoms, set the Amber10: EHT force field, and define the active sites of the proteins. The active sites were isolated for docking runs. The main components in the EOs were identified as those compounds with concentrations equal to or greater than 2% in the EO samples. The chemical structures of these compounds were downloaded from the PubChem database and their energies were minimized, with the appropriate AM1-BCC force field set using MOE software. The docking results of the compounds were evaluated based on docking scores and interactions with the active sites. Binding affinity (ΔG) was calculated using the Affinity dG function, which describes the change in free energy when the ligand interacts with the protein. The 10 lowest energy poses of each compound were saved for interaction analysis. Redocking of the co-crystallized ligand into the protein was performed to validate the docking method, with the RMSD value calculated. BOVIA Discovery Studio Visualizer and PyMOL software were used to visualize and analyze the interactions of the ligands with the active sites of the proteins.

## Results and Discussion

### Analysis of Essential Oils

The yields of hydrodistillation EO were from 0.01% to 0.12% aerial parts and leaves, respectively, as calculated on a fresh-weight basis (Table 1). The yields of hydrodistillation EOs extracted from the mangrove plants in the present study are similar to the EO yields by hydrodistillation of the same plants reported in previous investigations (0.05% to 0.17%) [35-38]. In literature, the yield of EOs obtained through hydrodistillation varies significantly (0.02-1.2%) depending on many factors, such as plant material, extraction conditions, and the use of additives [39-44]. Under optimal conditions, the yield of EOs obtained through hydrodistillation is improved remarkably and may be as much as 4.18-6.60% [45, 46].

The four EOs were yellowish. The chemical composition of EO samples was analyzed using gas chromatography coupled with mass spectrometry (Table S2). The concentration of each component was calculated based on the percentage of peak area in the total ion chromatogram. GC-MS analysis led to the identification of 50 volatile components in *A. glabra* EO (accounting for 98.18% of total oil), 41 volatile components in *S. trilobata* EO (accounting for 98.59% of total oil), 51 volatile compounds in *V. trifolia* EO (accounting for 97.49% of total oil), and 66 volatile compounds in *V. rotundifolia* EO (accounting for 94.4% of total oil). The main compounds were *β*-caryophyllene (26.45% in *A. glabra* EO, 66.25% in *V. trifolia* EO), *α*-pinene (37.65% in *S. trilobata* EO), and sclarene (15.05% in *V. rotundifolia* EO). There were various monoterpenes identified in all of four EOs, including *α*-thujene (0.09-0.24%), *α*-pinene (0.95-37.65%), sabinene (0.08-10.58%), *β*-pinene (0.15-2.36%), *β*-myrcene (0.18-5.38%), *α*-terpinene (0.05-0.68%), limonene (0.31-6.77%), and terpinolene (0.08-0.23%). Moreover, some monoterpenoids, including terpinen-4-ol (0.06-1.65%) and *γ*-terpineol (0.07-0.29%), and sesquiterpenoids, including *α*-cadinol (0.26-0.93%) and epi-*γ*-eudesmol (0.10-0.88%), were also found in all of the studied EOs, but in quite low contents. Sesquiterpenes, including *β*-caryophyllene (0.47-66.25%), alloaromadendrene (0.10-2.90%), *γ*-cadinene (0.14-1.77%), and zonarene (0.11-1.87%) were also similar compounds in 4 EOs.

The EO composition of the four species was compared to that of these species in previous studies. The EO of *A. glabra* from Brazil showed the ascendant content of *α*-pinene (11.3-18.5%), limonene (20.0-20.7%), *α*-phellandrene (1.20-21.0%), and (E)-*β*-ocimene (15.8-19.7%) [47]. Meanwhile, the EO from *A. glabra* collected in Cuba possessed high contents of myrcene (47.1%), (Z)-*β*-ocimene (16.3%), limonene (11.2%), and *α*-pinene (9.5%) [48]. Another study in Vietnam showed that the main components of *A. glabra* EO were *β*-caryophyllene (21.5%), germacrene D (17.7%), *α*-cardiol (5.4%), *β*-elemene (5.2%), *α*-phellandrene (4.3%), and *α*-caryophyllene (3.6%) [21]. These results suggested that *α*-pinene, limonene, and *α*-phellandrene were characteristic compounds in *A. glabra* EO. However, there are differences in the proportions of other compounds. Sesquiterpenes are the dominant compounds in the EO of *A. glabra* in Vietnam, while monoterpenoids are the dominant compounds in plants from Brazil and Cuba.

The EO from the flowers of *S. trilobata* reported from China has *α*-phellandrene (28.8%), germanacrene D (15.7%), and limonene (14.2%) as the main components [35]. According to another study from Brazil, *α*-pinene (30.3%), *α*-phellandrene (17.4%), *β*-pinene (6.4%), limonene (16.3%), and *γ*-muurolene (5.3%) are the main components in the EO of this species [36]. There are differences in the percentage of the main components compared to the studies from China and Brazil; however, *α*-pinene and *α*-phellandrene are the compounds with the highest proportions and they are consistent with the results of the study.

The EO of *V. trifolia* has been previously studied in different geographical locations, such as India and Vietnam, and reports have shown that the main components of the EO are *β*-caryophyllene (8.9–38.36%), sabinene (9.4–19.4%), and 1,8-cineol (15.7–25.7%) [37, 49, 50] Compared to the above results, the compounds *β*-caryophyllene, sabinene, and 1,8-cineol are all present in the EO of *V. trifolia* studied, with *β*-caryophyllene accounting for a very high proportion compared to the average proportion from other studies (66.25%), while eucalyptol accounts for a modest proportion (0.5%).

Research in Korea on the EO of *V. rotundifolia* has shown that the main components of this species are *α*-terpineol (13.1%), manool oxide (14.3%), and *α*-pinene (10.0%) [38]. Another report, also from Korea, suggests that *α*-pinene (13.24-30.25%), *β*-pinene (2.39-9.79%), and 1,8-cineol (4.40-19.89%) are the main components of this EO [51]. However, a report from Vietnam has shown that the prominent components of the EO of *V. rotundifolia* include sclareol (29.01%), sandaracopimarinal (16.51%), abieta-7,13-diene (15.65%), and verticillol (4.89%) [52]. Components such as *α*-pinene and *β*-pinene are present in the study in significant amounts (13.57% and 2.56%, respectively); however, the reported results have shown that the chemical composition of *V. rotundifolia* in Vietnam is mainly concentrated in diterpenes and diterpenoids as compared to monoterpenes in Korean report, and this is consistent with the results of the study.

### Antimicrobial Activity of Essential Oils against *S. aureus*

From the results in Table 3, the EOs of *A. glabra* and *S. trilobata* had antimicrobial effects on *S. aureus*, with *S. trilobata* exhibiting bactericidal effects with MIC and MBC values of 0.2%, while *A. glabra* exhibited bacteriostatic effects with MIC and MBC values of 0.8%. The two species *A. glabra* and *S. trilobata* can act as bactericidal antibiotics and have a ratio of MBC/MIC = 1. However, the EO of *V. rotundifolia* showed no inhibitory or bactericidal effects on *S. aureus* up to 0.8% EO. *V. trifolia* EO exhibited bactericidal effects with MIC values of 0.8%, and showed no bactericidal effects at a concentration of 0.8% EO. The antibacterial activity of the EOs extracted from these mangrove plants against MRSA is reported for the first time in the present study.

To date, there is no report on the antimicrobial activity of *A. glabra* EO, whereas the investigations of the antimicrobial activity of EOs extracted from the three mangrove plants *S. trilobata*, *V. trifolia*, and *V. rotundifolia* are still limited. The *S. trilobata* EO was reported showing antimicrobial activity against *S. aureus*, *Bacillus subtilis* (MIC = 2 mg/ml), *Propionibacterium granulosum* (MIC = 595 ± 206 μg/ml and MBC = 1,191 ± 413 μg/ml), *Escherichia coli*, *Pseudomonas aeruginosa*, *Epidermophyton floccosum* and *Trichophyton rubrum* with zones of inhibition reaching up to 17.73 mm [53-56]. The *V. trifolia* EO was found to exhibit antibacterial activity against *S. aureus* and antifungal activity against *Candida krusei* and *C. kefyr* with caryophyllene and *α*-terpineol identified as significant inhibitors in molecular docking studies [57, 58]. Other investigations indicate antimicrobial activity of *V. rotundifolia* EO against *S. aureus*, *B. cereus*, *P. aeruginosa*, *S. enterica* ser. Enteritidis, *S. enterica* ser. Typhimurium, *E. coli* (inhibition zones from 8.3 ± 0.3 to 27.3 ± 0.6 mm), *P. acnes*, and *S. epidermidis* (MIC = 62.5 – 2,000 μg/ml) [52, 59].

The antibacterial properties of EOs in the present study may be related to the content of the monoterpene compounds in the EOs, as different contents also affect the degree of expression of the inhibitory effect on *S. aureus*. In the investigated plants, *S. trilobata* EO contained a much higher content of monoterpene compounds than the other three species, and the antibacterial activity of its EO against *S. aureus* is also much better than that of the other EOs (MIC = 0.2-0.8%). Previous investigations suggest that monoterpenes are significant contributors to the antimicrobial activity of EOs, exerting such activity against various bacterial strains, including multidrug-resistant *S. aureus* [60-62]. Their effectiveness is attributed to their ability to disrupt bacterial cell membranes [62], inhibit biofilm formation [61], and act as efflux pump inhibitors [61, 63], which are crucial in overcoming antibiotic resistance.

### Docking Study of Essential Oil Components to the Relevant Targets of *S. aureus*

To gain a clearer understanding of the antibacterial activity of EOs at the molecular level, a molecular docking study was conducted to investigate the interactions between the main components (in this research, the main components >2%) in the EOs and target proteins of *S. aureus*. The docking method was validated by redocking the co-crystallized ligands into the protein crystal structure. The redocking results are shown in Table 4, indicating that all four ligands had an RMSD of less than 2 Å compared to their original conformations. This validated that the docking simulation method used is accurate and can be employed for experimental predictions [64, 65].

The binding energy results indicate that most of the compounds are capable of interacting with the active sites of key *S. aureus* proteins, as evidenced by their negative free binding energies (Table 5). Among these, the compounds showed the highest binding affinity with dihydrofolate reductase (PDB ID: 2W9S). Interestingly, most of the main components in the EOs had better docking scores than the co-crystallized ligand (trimethoprim, TMP), underscoring the inhibitory effect of these compounds on this enzyme.

**Fig. 1:**
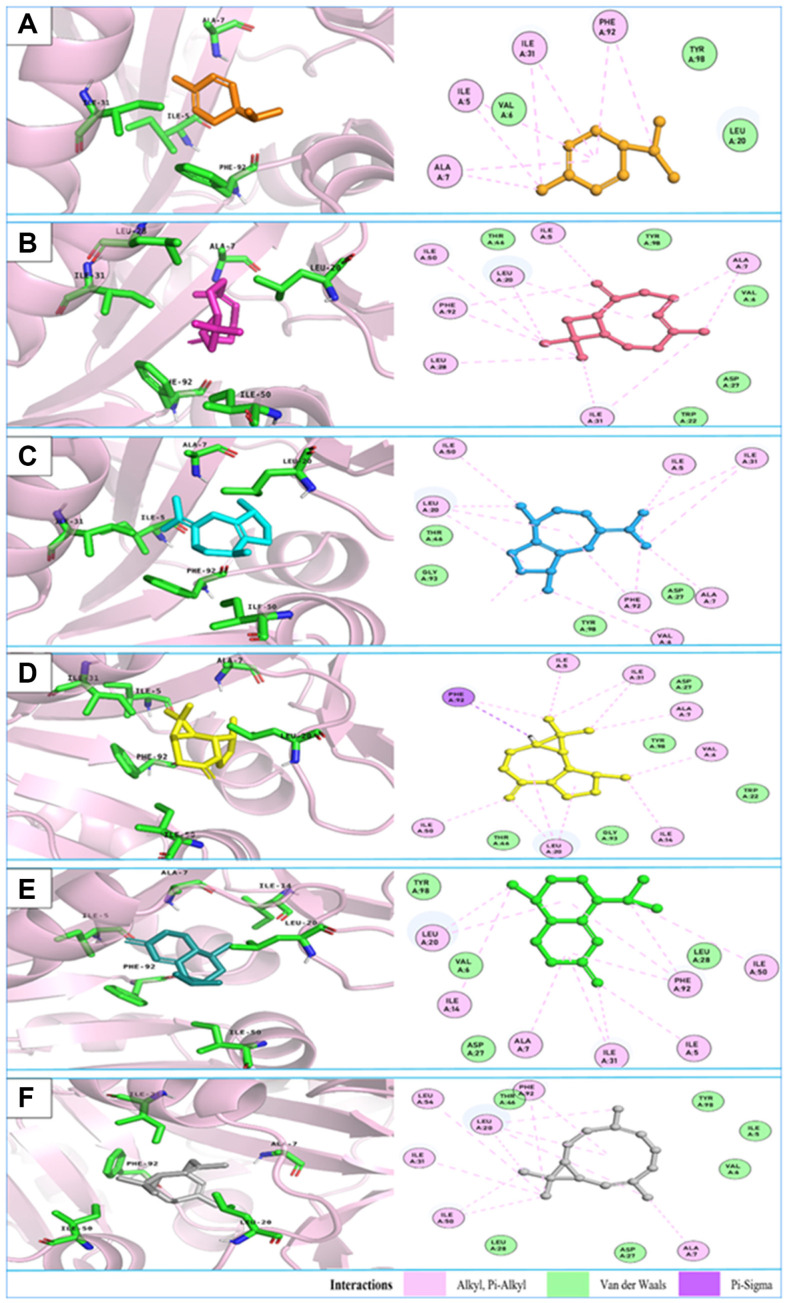
3D and 2D interactions of the six best binding compounds with *S. aureus* DHFR: (**A**) α-phellandrene; (**B**) β-caryophyllene; (**C**) β-guaiene; (**D**) alloaromadendrene; (**E**) bicyclosesquiphellandrene; (**F**) bicyclogermacrene.

Dihydrofolate reductase (DHFR) is a critical enzyme for most microorganisms, catalyzing the reduction of dihydrofolate (DHF) to tetrahydrofolate (THF), which is an essential metabolite for microbial growth and replication. Numerous studies have demonstrated that inhibiting DHFR is a promising approach for treating bacterial infections [66, 67]. Notably, DHFR consists of two isozymes: TMP-sensitive and TMP-resistant enzymes, encoded by different genes. The protein crystal 2W9S selected for this study is encoded by a mutant gene and is the TMP-resistant isozyme that has emerged in MRSA infections [32, 66]. Docking results show that all major components in the EOs had significant binding energies to DHFR, ranging from approximately -5 kcal/mol to -7 kcal/mol (Table 5). Among the compounds studied, six exhibited particularly high binding energies (greater than -7 kcal/mol): *α*-phellandrene, *β*-caryophyllene, *β*-guaiene, alloaromadendrene, bicyclosesquiphellandrene, and bicyclogermacrene. These compounds all interacted with the enzyme's folate pocket (Fig. 1) and formed crucial hydrophobic interactions with amino acids Phe92 and Leu20 [32]. Additionally, they also formed hydrophobic interactions with Ile50, Val6, Ile31, Ile5, and Ala7 in the active site (Fig. 1). Among these, *α*-phellandrene, *β*-caryophyllene, bicyclosesquiphellandrene, and bicyclogermacrene are the main components in the *S. trilobata* EO; *β*-caryophyllene, *β*-guaiene, and alloaromadendrene are the main components in the *A. glabra* EO; and *β*-caryophyllene and alloaromadendrene are the main components in the *V. trifolia* EO (Table 2). Therefore, these docking results help in elucidating the role of the main chemical components in the EOs and their antibacterial effects against *S. aureus*. The high affinity of these compounds for the drug-resistant isozyme further suggests the potential of these EOs to combat drug-resistant *S. aureus*.

## Conclusion

In the present study, we investigated the antibacterial activity of essential oils from four medicinal plants collected in the Xuan Thuy mangrove forest. These plants include *S. trilobata*, *V. rotundifolia*, *V. trifolia*, and *A. glabra*. The essential oil yields from hydrodistillation of aerial parts and leaves varied between 0.01% to 0.12%v/w based on fresh weight. Subsequently, *in vitro* studies revealed that essential oils from *A. glabra*, *S. trilobata*, and *V. trifolia* exhibited antibacterial properties against *S. aureus* with MIC and MBC values of 0.2-0.8% EO. Conversely, *V. rotundifolia* essential oil did not inhibit *S. aureus* even at concentrations up to 0.8% EO. Furthermore, docking analysis identified six main compounds with notably binding energies (< -7 kcal/mol), including *α*-phellandrene, *β*-caryophyllene, *β*-guaiene, alloaromadendrene, bicyclosesquiphellandrene, and bicyclogermacrene. These findings highlight the antibacterial potential of EOs from mangrove plants; therefore, expanded investigations into EOs and the biological properties from mangrove plants may be a promising strategy for combating antimicrobial resistance.

## Supplemental Materials

Supplementary data for this paper are available on-line only at http://jmb.or.kr.



## Figures and Tables

**Table 1:** EO samples of four medicinal plants.

ID	Scientific name	Voucher specimen code	Plant parts	Essential oil extraction		
				Weight of material (g)	Volume of oil (ml)	Oil yield (v/m)^a^
1	*A. glabra*	XT14	Leaves	900	0.5	0.05%
2	*V. rotundifolia*	XT48	Leaves	1600	0.65	0.04%
3	*V. trifolia*	XT16	Leaves	1000	1.3	0.12%
4	*S. trilobata*	XT30	Aerial parts	1000	0.16	0.01%

^a^Calculated on fresh materials

**Table 2:** Volatile main compounds identified in essential oils of *A. glabra*, *S. trilobata*, *V. trifolia*, *V. rotundifolia*.

No.	Compounds	RI^cal^	RI^lit^	Classification	Relative Abundance %			
					AG	ST	VT	VR
	*α*-Pinene	936	932	Monoterpene	3.13	37.65	0.95	13.57
	Sabinene	976	974	Monoterpene	0.08	0.94	10.58	3.94
	*β*-Pinene	978	979	Monoterpene	0.15	0.85	1.01	2.36
	*β*-Myrcene	994	991	Monoterpene	5.38	0.51	0.26	0.18
	*α*-Phellandrene	1007	1005	Monoterpene	7.28	22.62	0.10	-
	*p*-Cymene	1028	1025	Monoterpene	0.57	2.28	0.04	-
	Limonene	1032	1030	Monoterpene	3.44	6.77	0.31	0.62
	*β*-*trans*-Ocimene	1051	1049	Monoterpene	5.72	0.18	-	-
	*β*-Caryophyllene	1429	1428	Sesquiterpene	26.45	2.11	66.25	0.47
	Alloaromadendrene	1460	1461	Sesquiterpene	2.90	0.21	2.04	0.10
	Bicyclosesquiphellandrene	1488	1489	Sesquiterpene	-	3.79	0.73	0.74
	*β*-Guaiene	1490	1490	Sesquiterpene	24.16	-	-	-
	Bicyclogermacrene	1503	1500	Sesquiterpene	0.72	7.10	-	-
	Phenylheptatriyne	1713	1725	Aromatic	-	2.25	-	-
	Isovalencenyl formate	1802	1800	Ester	-	-	-	4.21
	Rimuene	1891	1896	Diterpene	-	-	1.95	5.86
	Verrucarol	1939	1939	Sesquiterpenoid	-	-	-	3.33
	*p*-Anilinophenol	1953	1956	Aromatic	-	-	-	2.19
	Isophyllocladene	1962	1966	Diterpene	-	-	1.79	5.32
	Sclarene	1973	1974	Diterpene	0.05	-	0.16	15.05
	13-epi-Dolabradiene	2000	2000	Diterpene	-	-	-	8.08
	Levopimaradiene	2033	2040	Diterpene	-	-	-	8.03
	Verticillol	2097	2106	Diterpenoid	-	-	0.06	6.32

“-“ – Not detected; AG - *Annona glabra*, ST – *Sphagneticola trilobata*, VT – *Vitex trifolia*, VR – *Vitex rotundifolia*

**Table 3:** *In vitro* antibacterial activity of EOs against *Staphylococcus aureus*.

Concentration (%, v/v)	Essential oil samples			
	*A. glabra*	*S. trilobata*	*V. trifolia*	*V. rotundifolia*
MIC	0.8	0.2	0.8	>0.8
MBC	0.8	0.2	>0.8	>0.8

**Table 4:** Validation results of the docking method by redocking.

PDB ID	Protein	Co-crystallized ligand	RMSD (Å)
1JIJ	Tyrosyl-tRNA synthetase	SB-239629	1.798
2W9S	Dihydrofolate reductase	Trimethoprim	0.749
2ZCQ	Dehydrosqualene synthase	BPH-652	0.856
4DXD	Cell division protein FtsZ	PC190723	0.204

**Table 5:** Binding energies (kcal/mol) calculated from molecular docking results.

Compounds	Proteins of *Staphylococcus aureus*			
	1JIJ	2W9S	2ZCQ	4DXD
*α*-Pinene	-3.53	-5.73	-3.87	-3.98
*α*-Phellandrene	-3.82	**-7.09**	-5.81	-4.50
*β*-Caryophyllene	-4.06	**-7.36**	-4.42	-4.38
*β*-Guaiene	-4.31	**-7.15**	-4.14	-4.57
*β*-Myrcene	-3.59	-5.50	-5.18	-4.27
*β*-Pinene	-3.24	-5.66	-5.42	-4.16
*β*-*trans*-Ocimene	-3.90	-5.63	-4.70	-4.26
Alloaromadendrene	-3.72	**-7.21**	-5.20	-4.40
Bicyclosesquiphellandrene	-4.06	**-7.24**	-4.62	-4.95
Bicyclogermacrene	-4.34	**-7.42**	-5.15	-4.43
Isovalencenyl formate	-4.09	-5.12	-5.73	-4.58
Isophyllocladene	-3.98	-5.58	-5.91	-4.90
Levopimaradiene	-4.57	-5.61	-5.92	-4.71
Limonene	-3.69	-5.64	-6.03	-4.48
*p*-Anilinophenol	-4.96	-6.45	-5.48	-4.39
*p*-Cymene	-3.68	-5.60	-5.02	-4.57
Phenylheptatriyne	-4.12	-6.63	-6.47	-5.04
Rimuene	-4.44	-6.83	-5.86	-5.05
Sabinene	-3.56	-5.63	-5.32	-4.53
Sclarene	-4.59	-6.84	-5.67	-5.31
Verrucarol	-4.36	-5.08	-5.41	-5.19
Verticillol	-4.89	-6.28	-4.15	-4.70
13-*epi*-Dolabradiene	-4.20	-6.69	-5.02	-5.12
**Co-crystallized ligand**	-6.41	-6.49	**-8.07**	-6.00
