# Supplementary material for: In Vitro and In Silico Antimicrobial Activity against Methicillin-Resistant Staphylococcus aureus of Essential Oils from Four Medicinal Plants in Xuan Thuy Mangrove Forest
Source: J Microbiol Biotechnol. 2025 Feb 14;35:e2409006. doi: 10.4014/jmb.2409.09006 (PMC11876009; doi:10.4014/jmb.2409.09006)
Supplement: Supplementary file 1 [file jmb-35-e2409006-supple.pdf]

J. Microbiol. Biotechnol. 2025. 35: e2409006
https://doi.org/10.4014/jmb.2409.09006

Supplementary Tables

In vitro and *In silico* antimicrobial activity against methicillin-resistant *Staphylococcus aureus* of essential oils from four medicinal plants in Xuan Thuy mangroves forest

Ngoc Anh Luu Dam[1*], Van Huong Bui[1*], Huu Dat Ton That[2], Khac Tiep Nguyen[3], Thanh Tung Nguyen[3], Quang Quy Duong[3], Tung Lam Vo[3], and Vladimir V. Titok[4]

[1]*Vietnam National Museum of Nature, Vietnam Academy of Science and Technology, 18 Hoang Quoc Viet, Cau Giay, Ha Noi, Vietnam*

[2]*Mientrung Institute for Scientific Research, Vietnam National Museum of Nature, Vietnam Academy of Science and Technology, 321 Huynh Thuc Khang, Hue City, Vietnam*

[3]*Hanoi University of Pharmacy, 13-15 Le Thanh Tong, Hoan Kiem, Ha Noi, Vietnam*

[4]*Central Botanical Garden, National Academy of Sciences of Belarus, Minsk, Belarus*

**Corresponding authors: ngocanh@vnmn.vast.vn*

Table S1. Volatile compounds identified in essential oils of *A. glabra, S. trilobata, V. trifolia, V. rotundifolia.*

No.	Compounds	RI^{cal}	RI^{lit}	Classification	Relative Abundance %			
					AG	***ST***	***VT***	***VR***
1	Tricyclene	923	925	Monoterpene	-	0.06	-	-
2	*α*-Thujene	929	929	Monoterpene	0.09	0.16	0.24	0.23
3	*α*-Pinene	936	932	Monoterpene	3.13	37.65	0.95	13.57
4	Camphene	950	952	Monoterpene	1.49	1.39	-	0.10
5	2,4(10)-Thujadiene	955	956	Monoterpene	-	0.05	-	-
6	Sabinene	976	974	Monoterpene	0.08	0.94	10.58	3.94
7	*β*-Pinene	978	979	Monoterpene	0.15	0.85	1.01	2.36
8	1-Octen-3-ol	985	980	Alcol	-	-	0.35	0.08
9	3-Octanone	990	986	Cetone	-	-	0.23	-
10	*β*-Myrcene	994	991	Monoterpene	5.38	0.51	0.26	0.18
11	3-Octanol	1001	994	Alcol	-	-	0.05	-
12	2-Carene	1004	1001	Monoterpene	0.05	-	-	-
13	*α*-Phellandrene	1007	1005	Monoterpene	7.28	22.62	0.10	-
14	*α*-Terpinene	1019	1017	Monoterpene	0.05	0.18	0.68	0.17
15	*o*-Cymene	1022	1022	Monoterpene	0.05	-	-	-
16	*p*-Cymene	1028	1025	Monoterpene	0.57	2.28	0.04	-
17	Limonene	1032	1030	Monoterpene	3.44	6.77	0.31	0.62
18	1,8-Cineole	1034	1032	Monoterpenoid	-	-	0.50	0.20
19	*β*-*cis*-Ocimene	1041	1038	Monoterpene	0.27	0.70	-	-
20	*β*-*trans*-Ocimene	1051	1049	Monoterpene	5.72	0.18	-	-
21	*γ*-Terpinene	1062	1060	Monoterpene	0.07	0.09	0.94	0.27

No.	Compounds	RI^{cal}	RI^{lit}	Classification	Relative Abundance %			
					AG	*ST*	*VT*	*VR*
22	*trans*-4-Thujanol	1072	1070	Monoterpenoid	-	-	0.11	-
23	Terpinolene	1091	1088	Monoterpene	0.11	0.10	0.23	0.08
24	Perillene	1105	1101	Monoterpenoid	0.10	-	0.12	0.11
25	2-Nonen-1-ol	1107	1105	Alcol	-	-	0.05	-
26	*cis*-2-p-Menthen-1-ol	1127	1122	Monoterpenoid	-	-	0.10	0.07
27	*trans*-p-2-Menthen-1-ol	1145	1140	Monoterpenoid	-	-	0.05	0.10
28	*Z*-Isocitral	1154	1160	Monoterpenoid	-	0.08	-	-
29	*dl*-Isopulegol	1156	1163	Monoterpenoid	-	-	-	0.07
30	Pinocarvone	1166	1164	Monoterpenoid	-	-	-	0.08
31	Ocimenol	1177	1174	Monoterpenoid	-	-	0.14	0.05
32	Terpinen-4-ol	1183	1182	Monoterpenoid	0.06	0.17	1.65	0.51
33	*γ*-Terpineol	1197	1197	Monoterpenoid	0.07	0.09	0.29	0.10
34	Shisool	1290	1291	Monoterpenoid	-	-	-	0.24
35	Dihydroedulan	1293	1293	Sesquiterpene	-	-	0.16	0.07
36	Silphiperfol-5-ene	1330	1331	Sesquiterpene	-	-	-	0.22
37	*δ*-EIemene	1342	1338	Sesquiterpene	0.39	-	-	-
38	7-epi-Silphiperfol-5-ene	1349	1348	Sesquiterpene	-	-	-	1.62
39	*α*-Cubebene	1354	1351	Sesquiterpene	0.10	-	-	0.39
40	Silphiperfola-4,7(14)-diene	1362	1362	Sesquiterpene	-	-	-	0.07
41	Copaene	1381	1376	Sesquiterpene	1.31	-	0.05	0.24
42	Daucene	1389	1381	Sesquiterpene	0.30	-	-	0.06
43	*β*-Cubebene	1395	1389	Sesquiterpene	0.96	0.19	-	-

No.	Compounds	RI^{cal}	RI^{lit}	Classification	Relative Abundance %			
					AG	*ST*	*VT*	*VR*
44	*β*-Elemene	1397	1391	Sesquiterpene	0.30	0.10	-	0.14
45	Isoitalicene	1399	1395	Sesquiterpene	-	-	0.23	-
46	*cis*-Caryophyllene	1413	1406	Sesquiterpene	-	-	0.05	-
47	*α*-Cedrene	1421	1411	Sesquiterpene	0.17	-	-	-
48	*β*-Caryophyllene	1429	1428	Sesquiterpene	26.45	2.11	66.25	0.47
49	Aromandendrene	1440	1440	Sesquiterpene	0.84	-	-	-
50	Cadina-3,5-diene	1456	1458	Sesquiterpene	-	0.06	-	-
51	Alloaromadendrene	1460	1461	Sesquiterpene	2.90	0.21	2.04	0.10
52	*α*-Elemene	1467	1462	Sesquiterpene	0.21	-	-	-
53	*cis*-Muurola-4(15),5-diene	1470	1463	Sesquiterpene	0.05	-	-	-
54	*trans*-Cadina-1(6),4-diene	1479	1475	Sesquiterpene	-	0.12	-	-
55	*γ*-Muurolene	1484	1478	Sesquiterpene	0.18	-	-	-
56	Bicyclosesquiphellandrene	1488	1489	Sesquiterpene	-	3.79	0.73	0.74
57	*β*-Guaiene	1490	1490	Sesquiterpene	24.16	-	-	-
58	*epi*-Cubebol	1498	1493	Sesquiterpenoid	-	0.34	-	0.10
59	*γ*-Amorphene	1500	1496	Sesquiterpene	0.43	-	-	-
60	*α*-Selinene	1502	1498	Sesquiterpene	-	-	0.08	0.06
61	Bicyclogermacrene	1503	1500	Sesquiterpene	0.72	7.10	-	-
62	*trans*-*β*-Guaiene	1506	1502	Sesquiterpene	0.53	-	-	0.06
63	*γ*-Cadinene	1512	1509	Sesquiterpene	1.77	0.40	0.14	0.16
64	*β*-Cadinene	1521	1513	Sesquiterpene	0.67	0.09	-	0.06
65	*δ*-Cadinene	1525	1524	Sesquiterpene	-	0.81	-	0.09
66	Zonarene	1530	1528	Sesquiterpene	1.61	1.87	0.11	1.29

No.	Compounds	RI^{cal}	RI^{lit}	Classification	Relative Abundance %			
					AG	*ST*	*VT*	*VR*
67	*trans*-*γ*-Bisabolene	1537	1533	Sesquiterpene	0.42	-	-	-
68	Elemicin	1563	1555	Aromatic	-	-	0.13	0.26
69	Germacrene B	1565	1559	Sesquiterpene	0.66	-	-	0.10
70	Ledol	1570	1565	Sesquiterpenoid	-	-	-	0.08
71	Zierone	1584	1574	Sesquiterpenoid	-	-	0.23	0.17
72	*ar*-Tumerol	1586	1582	Sesquiterpenoid	0.06	0.33	0.08	-
73	*β*-Copaen-4α-ol	1588	1586	Sesquiterpenoid	-	1.80	-	-
74	Isoaromadendrene epoxide	1592	1589	Sesquiterpenoid	0.37	-	1.46	-
75	Guaiol	1607	1600	Sesquiterpenoid	-	0.07	-	-
76	Khusimone	1609	1605	Sesquiterpenoid	-	-	0.08	-
77	1-*epi*-Cubenol	1632	1627	Sesquiterpenoid	1.87	-	-	0.16
78	*α*-*epi*-Cadinol	1637	1640	Sesquiterpenoid	-	0.64	-	0.11
79	*τ*-Cadinol	1642	1640	Sesquiterpenoid	0.20	-	-	-
80	*α*-Cadinol	1653	1653	Sesquiterpenoid	0.93	0.54	0.64	0.26
81	Isoelemicin	1658	1654	Aromatic	0.22	-	-	-
82	*epi*-*γ*-Eudesmol	1666	1662	Sesquiterpenoid	0.88	0.64	0.10	0.22
83	Phenylheptatriyne	1713	1725	Aromatic	-	2.25	-	-
84	Methyl zizanoate	1740	1739	Ester	-	-	-	0.06
85	Drimenol	1754	1761	Sesquiterpenoid	-	-	-	0.48
86	Isovalencenyl formate	1802	1800	Ester	-	-	-	4.21
87	*trans*-Valerenyl acetate	1842	1832	Ester	-	-	-	0.09
88	8S,14-Cedrandiol	1871	1876	Sesquiterpenoid	-	-	0.12	-
89	Rimuene	1891	1896	Diterpene	-	-	1.95	5.86

No.	Compounds	RI^{cal}	RI^{lit}	Classification	Relative Abundance %			
					AG	*ST*	*VT*	*VR*
90	Isopimara-9(11),15-diene	1898	1905	Diterpene	-	-	0.45	0.74
91	Biformene	1921	1932	Diterpene	-	-	-	0.29
92	Cembrene	1937	1937	Diterpene	-	-	-	1.49
93	Verrucarol	1939	1939	Sesquiterpenoid	-	-	-	3.33
94	*p*-Anilinophenol	1953	1956	Aromatic	-	-	-	2.19
95	Isophyllocladene	1962	1966	Diterpene	-	-	1.79	5.32
96	Sclarene	1973	1974	Diterpene	0.05	-	0.16	15.05
97	Manool oxide	1983	1987	Diterpenoid	-	-	-	0.65
98	13-epi-Dolabradiene	2000	2000	Diterpene	-	-	-	8.08
99	(S,E)-8,12,15,15-Tetramethyl-4-methylenebicyclo[9,3,1]pentadeca-7,11-diene	2024	2027	Diterpene	-	0.07	0.49	-
100	Levopimaradiene	2033	2040	Diterpene	-	-	-	8.03
101	*cis*-3,14-Clerodadien-13-ol	2055	2051	Diterpenoid	-	-	0.18	-
102	7-Isopropyl-1,1,4a-trimethyl-1,2,3,4,4a,9,10,10a-octahydrophenanthrene	2067	2074	Diterpene	-	-	-	1.45
103	Kolavelool	2083	2080	Diterpenoid	-	-	0.40	0.07
104	Abieta-7,13-diene	2093	2087	Diterpene	0.31	-	0.10	-
105	Verticillol	2097	2106	Diterpenoid	-	-	0.06	6.32
106	Agathadiol	2250	2266	Diterpenoid	-	-	0.25	0.26

No.	Compounds	RI^{cal}	RI^{lit}	Classification	Relative Abundance %			
					AG	***ST***	***VT***	***VR***
107	4-*epi*-Abietal	2277	2298	Diterpenoid	-	0.19	-	-
	**Total**				**98.18**	**98.59**	**97.49**	**94.40**

"-" – Not detected; AG - Annona glabra, ST – Sphagneticola trilobata, VT – Vitex trifolia, VR – Vitex rotundifolia
